# A Numerical Investigation on the Effect of Size and Volume Fraction of Red Blood Cells in a Microchannel with Sudden Expansion

**DOI:** 10.3390/mi17030316

**Published:** 2026-03-02

**Authors:** Cihan Sezer, Kenan Kaya, Mahdi Tabatabaei Malazi, Ahmet Selim Dalkılıç

**Affiliations:** 1 Department of Mechanical Engineering, Faculty of Mechanical Engineering, Yıldız Technical University, Istanbul 34349, Türkiye; dalkilic@yildiz.edu.tr; 2 Department of Mechanical Engineering, Faculty of Engineering, Istanbul Aydin University, Istanbul 34295, Türkiye; kenankaya@aydin.edu.tr (K.K.); mahditabatabaei@aydin.edu.tr (M.T.M.); 3 Scientific Research Department, Azerbaijan University of Architecture and Construction (AzUAC), Baku AZ1073, Azerbaijan

**Keywords:** Fahraeus–Lindqvist effect, microscale hemodynamics, multiphase flow, red blood cell distribution, cell-free layer, computational fluid dynamics

## Abstract

This study numerically investigates the effects of red blood cell (RBC) volume fraction (hematocrit) and RBC diameter on cell distribution, cell-free layer (CFL) thickness and pressure drop in a microchannel with sudden expansion. Hematocrit levels of 0.2, 0.3, 0.4 and 0.5, together with RBC diameters of 4, 8 and 11 µm, are considered, where deviations from the physiological diameter of 8 μm represent pathological conditions. An Euler–Euler approach is employed to model the multiphase flow, treating RBCs as rigid spherical particles, while the non-Newtonian viscosity of blood is represented using a modified Carreau–Yasuda model. The numerical predictions are validated against existing experimental and numerical data. The effect of volumetric flow rate on RBC distribution is found to be limited; therefore, a representative flow rate of 100 μL/min is adopted for the subsequent analysis. The results show that RBC migration and the resulting cell distribution are strongly governed by RBC size and hematocrit. The pressure drop is primarily influenced by hematocrit, while the effect of RBC size is relatively weak. A minimum value for pressure drop is observed at a hematocrit of 0.3, indicating an optimal hematocrit level for minimizing flow resistance. A parabolic correlation is proposed for predicting the pressure drop as a function of hematocrit, with a maximum relative error of 1.13%. This study contributes to the understanding of pathological RBC size variations and their impact on microscale hemodynamics.

## 1. Introduction

Red blood cells (RBCs) play a vital role in the human circulatory system since they are involved in oxygen supply to cells and tissues via a mass transfer mechanism across capillary walls. The Fahraeus–Lindqvist phenomenon attributes the decrease in dynamic blood viscosity in vessels with diameters below a critical threshold to the migration of RBCs from the near-wall region to the vessel core [1]. This migration causes formation of a cell-free layer (CFL) near the wall because of diminishing RBC concentration.

There is still no consensus among researchers regarding the exact mechanism responsible for the initiation of CFL formation in capillaries: Rodrigues et al. [2] claimed that the parabolic velocity profile of RBCs results in a high shear stress around the vessel wall that forces the RBCs to migrate towards the center. Gidaspow and Huang [3] stated that CFL formation depends on a quantity known as granular temperature, defined as the mean square of particle velocity fluctuations—a measure of the kinetic energy of RBCs per unit mass. According to kinetic theory, granular temperature is generated by viscous shear and is dissipated through inelastic particle collisions [4]. Gidaspow [5] further suggested that RBC migration can be explained by the Brownian motion of particles, as described by the kinetic theory of granular flow, which is based on Einstein’s classical work [6]. In contrast, Secomb and Pries [7] claimed that the Fahraeus–Lindqvist effect arises from deformability of RBCs. However, Kim et al. [8] and Gracka et al. [9] independently predicted CFL development in their numerical studies based on two main assumptions: (i) the granular temperature term was neglected in the Eulerian multiphase approach with a non-Newtonian viscosity model, and (ii) RBCs were treated as rigid spherical particles. These assumptions contrast with both Gidaspow’s kinetic theory-based explanation [5] and the deformability-based mechanism proposed by Secomb and Pries [7]. These interpretations indicate that the driving force behind CFL formation remains ambiguous.

A wide range of numerical approaches, including kinetic theory-based models, multiphase Euler–Euler formulations coupled with non-Newtonian viscosity models, the Volume of Fluid (VOF) method, the Discrete Phase Model (DPM), and the lattice Boltzmann method, have been employed to investigate multiphase blood flow in microchannels. Experimental methods have also been used to observe RBC motion and distribution in microfluidic systems. A summary of relevant studies on blood flow in microchannels is presented below and listed in Table 1.

Gidaspow and Huang [3] proposed a two-phase flow model based on kinetic theory, in which granular temperature was balanced to predict blood viscosity as a function of tube diameter and RBC volume fraction in narrow vessels. Kim et al. [8] reviewed mixture theory-based models that describe blood as a suspension of rigid spherical RBCs in plasma. In their analysis, interaction forces between RBCs and plasma, such as drag and shear lift, are considered, and a comparison of the drag force predictions using the Batchelor and Rourke–Erstene models is presented. They also included the spin lift effect, which is often neglected, and used two-phase flow simulations to predict RBC migration related to the Fahraeus–Lindqvist effect. Gracka et al. [9] studied blood flow in microchannels with hyperbolic contractions using both Euler–Euler and Euler–Lagrange approaches to model multiphase flow. Their CFD simulations predicted CFL thicknesses of about 5 μm and 35 μm for wide and narrow contractions, respectively. These results were validated experimentally, reducing the need for extensive prototype fabrication and laboratory testing. Soh et al. [10] introduced a CFD approach that incorporates multiphase and multicomponent flow physics using the Volume of Fluid (VOF) model. Their model tracked species transport using VOF-based smoothing, α-factor, and expulsion techniques. The model was shown to be applicable to a wide range of microscale flow problems, including pore-scale oil recovery, drug transport in blood vessels, and droplet dissolution. Jafari et al. [11] simulated blood flow in microvessels by employing a VOF-based computational model, which accounts for RBC motion and deformation in capillaries under complex flow conditions. Afzal and Kim [12] used non-Newtonian shear-dependent viscosity models to simulate blood flow in T-shaped and serpentine microchannels. While the Carreau–Yasuda model showed mixing performance comparable to that of a Newtonian fluid model, the serpentine microchannel exhibited superior mixing efficiency relative to the T-shaped geometry. Barbosa et al. [13] validated a DPM-based CFD model for blood flow through hyperbolic contractions, in which the RBC field was predicted. Their results showed strong agreement with experimental observations, with errors below 10%, enabling the optimization of microchannel design. Yin et al. [14] studied multiphase blood flow in a symmetric microvascular bifurcation numerically, using an immersed-boundary lattice Boltzmann method. They examined the CFL formation, RBC trajectories, phase separation, and RBC distribution while accounting for RBC deformability, aggregation, and inlet hematocrit. Their results showed that phase interactions strongly affect individual RBC trajectories, whereas phase separation is mainly governed by CFL thickness. Their analysis of the flow and pressure fields also demonstrated that shear stress influences cell sliding near the vessel walls, while pressure gradients drive bulk RBC motion across the microchannel. Yaginuma et al. [15] investigated RBC deformation in microfluidic devices with hyperbolic contraction followed by sudden expansion. They showed that RBC deformation reaches a plateau under constant extensional strain rates, while the plateau magnitude depends on the extension rate. According to their findings, microchannels with hyperbolic contraction can be effectively used to characterize RBC deformability for diagnostic and therapeutic applications. Based on their experimental work incorporating high-speed video microscopy, Pinho et al. [16] reported that temperature plays a significant role in microscale blood flow, which affects radial RBC distribution. Patrick et al. [17] investigated near-wall RBC motion in high-hematocrit blood by means of fluorescent dye labeling, time-resolved scanning confocal microscopy and micro-particle image velocimetry (μPIV). They observed intermittent flow behavior associated with cell elongation, localized flow obstructions, and transient rouleaux formation, validating the accuracy of confocal μPIV post-processing methods. Zhao et al. [18] studied microscale blood flow in prosthetic blood-contacting devices and tracked platelet-sized fluorescent particles in rapidly expanding RBC suspensions. They reported that hematocrit has a strong effect on RBC trajectories and distribution. For hematocrit values of 0.20 or lower, RBC-depleted near-wall regions larger than the RBC size were observed. Their work also indicated that rapid expansion can locally increase platelet concentration due to the redistribution of RBCs, indicating the indirect role of RBC dynamics in platelet transport. Lee et al. [19] experimentally developed a hyperbolic converging microchannel to evaluate RBC deformability in extensional flow fields, showing that extensional flows are more effective than shear flows in inducing deformation and highlighting the limitations of existing models. In addition, their approach was shown to be capable of identifying heat-induced variations in RBC deformability, which may improve the understanding of disease-related changes in RBC mechanical behavior under extensional flow conditions. Rashidi et al. [20] investigated the effect of RBC deformability on RBC distribution and CFL formation in a bifurcating microfluidic T-junction. Their experimental and numerical results showed that deformable RBCs generate the CFL more rapidly than rigid RBCs. Suwannaphan et al. [21] demonstrated that shear and extensional stresses induced by contraction–expansion microchannel geometries significantly affect RBC behavior in microscale blood flows. Recktenwald et al. [22] carried out experimental and numerical studies to investigate CFL formation in microchannels under steady and unsteady flow conditions. Their findings indicated that steady-flow formulations are able to capture the mean RBC behavior, whereas unsteady flow mainly affects its temporal modulation in microchannels. Kang [23] demonstrated that pulsatile microfluidic flows can be used to measure biomechanical properties of RBCs, including viscoelasticity and aggregation. Finally, Manekar et al. [24] combined CFD and machine learning to optimize blood–plasma separation, achieving plasma yields of approximately 90–95% and reducing design iteration time for point-of-care diagnostic applications.

Despite extensive experimental and numerical research on RBC migration, CFL development, and blood rheology in microchannels, the effects of disease-related alterations in RBC morphology on microscale hemodynamics have not been thoroughly investigated, to the best of our knowledge. Previous studies have shown that a variety of pathological conditions can significantly alter RBC size and shape, deviating from their nominal biconcave geometry and the typical diameter of approximately 8 µm [25]. Conditions such as microcytic hypochromic anemia, which is often associated with chronic blood loss, and hereditary spherocytosis are characterized by smaller and more spherical RBCs [26]. In contrast, megaloblastic anemia caused by vitamin B12 or folic acid deficiency, as well as diseases such as alcoholic liver disease and asthmatic bronchitis, are commonly associated with enlarged and irregularly shaped RBCs [26,27]. Systematic numerical investigations focusing on the effects of these morphological anomalies on microscale blood flow dynamics, especially in terms of RBC distribution, CFL formation, and flow resistance, are scarce.

To address this gap, the present study aims to isolate and quantify the effects of RBC size variations, representative of pathological morphological alterations, on microscale hemodynamics. Simulations are performed to investigate the combined effect of RBC diameter, hematocrit, and flow rate on RBC distribution, CFL thickness, and pressure drop (*ΔP*) in a microchannel with an abrupt expansion. Blood is modeled as a suspension of plasma and a dispersed RBC phase using a multiphase Euler–Euler framework. While the plasma phase is modeled as a Newtonian fluid with constant viscosity, the apparent non-Newtonian behavior of blood is described by assigning a shear- and concentration-dependent Carreau–Yasuda viscosity to the RBC phase. Pathological conditions are represented by considering RBC diameters of 4 and 11 µm, while 8 µm corresponds to the physiological condition. Hematocrit is systematically varied between 0.2 and 0.5. The findings provide new insights into pathological microcirculation and offer quantitative guidance for future studies on oxygen transport, drug delivery and the design of artificial microvascular systems and microfluidic diagnostic platforms, such as lab-on-a-chip devices, where RBC distribution, CFL thickness and pressure drop play a critical role.

## 2. Numerical Method

### 2.1. Model Assumptions

In the present numerical analysis, several assumptions are adopted to establish a two-phase blood flow model. The flow is assumed to be steady, laminar and incompressible, while gravitational and thermal effects are neglected. Plasma is treated as a Newtonian fluid with constant viscosity, whereas RBCs are modeled as a rigid, non-deformable dispersed phase represented by spherical particles. No mass transfer is allowed between the phases. The two-phase flow is described using an Euler–Euler framework, where the volume fractions of the plasma and RBC phases sum to unity at every point in the computational domain. These assumptions constitute the framework upon which the numerical model is constructed.

### 2.2. Two-Phase Blood Flow Model

**Figure 1:**
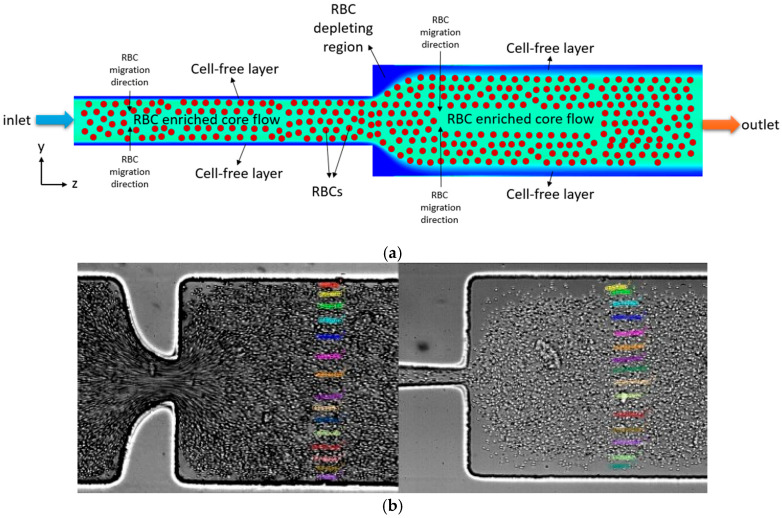
(**a**) Schematic illustration of RBC distribution and CFL formation in a microchannel with sudden expansion in this work. (**b**) RBC distribution and CFL structure in a microchannel with hyperbolic contraction [from Gracka et al. [9]].

Figure 1 illustrates the underlying physical configuration in this study. Two-phase flow is simulated using the Euler–Euler multiphase model, which relies on the fact that each point in space can be occupied solely by one of the phases. Hence, the sum of the volume fractions of the phases equals unity at each point in the domain, as shown in Equation (1). Throughout the text, the subscripts *p* and *R* stand for plasma and RBC phase, respectively.

(1) $$\alpha_{p}+\alpha_{R}=1$$

The discretized form of the three-dimensional incompressible steady-state Navier–Stokes equations, which include continuity and momentum equations given by Equations (2) and (3), respectively, are solved for each phase separately, using a commercial code, ANSYS^®^ Fluent 2023R2 [28]. The gravitational field is assumed to be negligible, while interphase drag (${\vec{F}}_{drag}$) and lift (${\vec{F}}_{lift}$) forces are included in the momentum equations as source terms. Constant densities of *ρ_p_* = 1027 kg/m^3^ and *ρ_R_* = 1093 kg/m^3^ are assumed for plasma and RBC phases, respectively [8].

Conservation of mass:

(2a) $$\nabla \cdot \left(\alpha_{p}{\vec{u}}_{p}\right)=0$$

(2b) $$\nabla \cdot \left({\alpha_{R}\vec{u}}_{R}\right)=0$$

Conservation of momentum:

(3a) $$\rho_{p}\nabla \cdot \left(\alpha_{p}{\vec{u}}_{p}{\vec{u}}_{p}\right)={-\alpha }_{p}\nabla p+\nabla \cdot {\overset{=}{\tau }}_{p}+{\vec{F}}_{drag,p}+{\vec{F}}_{lift,p}$$

(3b) $$\rho_{R}\nabla \cdot \left(\alpha_{R}{\vec{u}}_{R}{\vec{u}}_{R}\;\right)={-\alpha }_{R}\nabla p+\nabla \cdot {\overset{=}{\tau }}_{R}+{\vec{F}}_{drag,R}+{\vec{F}}_{lift,R}$$

Equations (1) and (2) constitute conservation of mass, as well as volume fraction equations. Convective terms in the transport equations of volume fraction (Equation (2)) and momentum (Equation (3)) are discretized using the first-order and second-order upwind schemes, respectively, while pressure is discretized using the PRESTO scheme. The pressure–velocity coupling is achieved using the coupled algorithm. The solution is initialized with zero RBC volume fraction throughout the domain, and the calculations are iterated until the RBC volume fraction profiles become invariant with successive iterations, indicating convergence of the phase distribution.

In this study, a rheological treatment for a shear-thinning non-Newtonian fluid within the generalized Newtonian framework is adopted, where the viscosity is a function of the shear rate solely, without introducing viscoelasticity effects. Therefore, the classical Newtonian representation of the constitutive stress relations is still valid as given by Equations (4a) and (4b):

(4a) $${\overset{=}{\tau }}_{p}=\alpha_{p}\mu_{p}\left(\nabla {\vec{u}}_{p}+\nabla {\vec{u}}_{p}^{T}\right)-\frac{2}{3}\alpha_{p}\mu_{p}\nabla \cdot {\vec{u}}_{p}\overset{=}{I}$$

(4b) $${\overset{=}{\tau }}_{R}=\alpha_{R}\mu_{R}\left(\nabla {\vec{u}}_{R}+\nabla {\vec{u}}_{R}^{T}\right)-\frac{2}{3}\alpha_{R}\mu_{R}\nabla \cdot {\vec{u}}_{R}\overset{=}{I}$$

It should be noted that the Stokes hypothesis is adopted in Equations (4a) and (4b), which allows the stress tensor to be expressed in terms of shear viscosity only. In Equation (4a), the plasma phase is treated as a Newtonian fluid, therefore, plasma viscosity $\mu_{p}$ is assumed constant (*μ_p_* = 0.00096 kg/m·s [8]). In contrast, whole blood exhibits shear-thinning behavior due to the presence of RBCs. To include this non-Newtonian characteristic of blood, a Carreau-Yasuda-type shear-dependent viscosity model proposed by Jung et al. [29] and Jung and Hassanein [30] is employed, where the dimensionless relative mixture viscosity η is given by Equation (5) as a function of the local shear rate and phase volume fractions:

(5) $$\eta =\frac{\alpha_{p}\mu_{p}+\alpha_{R}\mu_{R}}{\mu_{p}}=m{\left[1+{\left(\lambda \dot{\gamma }\right)}^{2}\right]}^{\left(n-1\right)/2}$$

Here, $\dot{\gamma }$ and *λ* denote the shear rate and the Carreau-Yasuda time constant, respectively, while *λ* is taken as 0.110 s [29,30,31]. The parameters *m* and *n* are correlation coefficients obtained by Jung and Hassanein [30] by curve-fitting of experimental blood viscosity to hematocrit for two distinct shear rate regimes:

For $\dot{\gamma }\geq 6$ s^−1^:

(6) $$m=122.28\alpha_{R}^{3}-51.213\alpha_{R}^{2}+16.305\alpha_{R}+1$$

(7) $$n=0.8092\alpha_{R}^{3}-0.8246\alpha_{R}^{2}-0.3503\alpha_{R}+1$$

For $\dot{\gamma }<6$ s^−1^:

(8) $$m=70.782\alpha_{R}^{3}-22.454\alpha_{R}^{2}+9.7193\alpha_{R}+1$$

(9) $$\frac{n-1}{k_{0}}=-0.8913\alpha_{R}^{3}+2.0679\alpha_{R}^{2}-1.7814\alpha_{R}$$

(10) $$k_{0}=\frac{\ln\left[\ln\left(1+{\left(\lambda \dot{\gamma }\right)}^{2}\right)\right]}{\ln\left(1+{\left(\lambda \dot{\gamma }\right)}^{2}\right)}$$

The effective RBC viscosity $\mu_{R}$ is calculated by rearranging the mixture relation in Equation (5):

(11) $$\mu_{R}=\frac{\mu_{p}\left(\eta -\alpha_{p}\right)}{\alpha_{R}}$$

To prevent numerical singularities, the effective mixture viscosity is constrained to reduce to the plasma viscosity as the RBC volume fraction approaches zero (*μ_mix_* → *μ_p_* as *α_R_* → 0).

Interphase drag (${\vec{F}}_{drag}$) and lift (${\vec{F}}_{lift}$) forces are considered as phase interaction forces, which are calculated by Equations (12) and (13), respectively.

(12) $${\vec{F}}_{drag}=\beta \left({\vec{u}}_{p}-{\vec{u}}_{R}\right)$$

(13) $${\vec{F}}_{lift}={-C}_{l}\rho_{p}\alpha_{R}\left({\vec{u}}_{p}-{\vec{u}}_{R}\right)\times \left(\nabla \times {\vec{u}}_{p}\right)$$

Here *β* and *C_l_* refer to the interphase momentum exchange coefficient and interphase lift coefficient, respectively. Gidaspow drag model [4], validated by Gidaspow and Huang [3] in microchannel blood flow simulations and recognized for its suitability in modeling microvascular hemodynamics, is used to calculate interphase momentum coefficient; which employs Ergun equation [32] when solid phase volume fraction is greater than 0.2, and Wen and Yu drag model [33] when solid phase volume fraction is less than 0.2. Although the minimum RBC volume fraction at the inlet is considered as 0.2 in this study, dilute mixture version of the drag model must also be considered because of RBC depletion near the wall region. The interphase momentum exchange coefficient *β* is calculated using the following equations, as suggested by Gidaspow [4]:

(14a) $$\beta =150\frac{\alpha_{R}^{2}\mu_{p}}{\alpha_{p}d_{R}^{2}}+1.75\frac{\rho_{p}\alpha_{R}\left|{\vec{u}}_{R}-{\vec{u}}_{p}\right|}{d_{R}}\;\;\;\alpha_{R}\geq 0.20$$

(14b) $$\beta =\frac{3}{4}C_{D}\frac{\alpha_{R}\alpha_{p}\rho_{p}\left|{\vec{u}}_{R}-{\vec{u}}_{p}\right|}{d_{R}}\alpha_{p}^{-2.65}\;\;\;\;\alpha_{R}<0.20$$

where *d_R_* represents the RBC diameter. In the Wen and Yu model (Equation (14b)), *C_D_* refers to drag coefficient and can be calculated by following:

(15) $$C_{D}=\frac{24}{\alpha_{p}{Re}_{R}}\left[1+0.15{\left(\alpha_{p}{Re}_{R}\right)}^{0.687}\right]$$

Here, *Re_R_* corresponds to particle Reynolds number which is based on plasma properties, RBC diameter and relative RBC velocity:

(16) $${Re}_{R}=\frac{\rho_{p}d_{R}\left|{\vec{u}}_{R}-{\vec{u}}_{p}\right|}{\mu_{p}}$$

Saffman [34] proposed a lift model for low Reynolds numbers (*Re_R_* < 40), which was later reviewed and extended by Mei and Klausner [35] to accommodate higher Reynolds numbers. Consequently, the Saffman-Mei lift model is employed to calculate the interphase lift coefficient, as shown in Equation (17).

(17) $$C_{l}=\frac{3}{2\pi \sqrt{{Re}_{\omega }}}C_{l}^{\prime }$$

(18a) $$C_{l}^{\prime }=6.46\;\left[\left(1-0.3314{\left(\frac{{Re}_{\omega }}{{2Re}_{R}}\right)}^{0.5}\right)e^{-0.1{Re}_{R}}+0.3314{\left(\frac{{Re}_{\omega }}{{2Re}_{R}}\right)}^{0.5}\right]\;{Re}_{R}\leq 40$$

(18b) $$C_{l}^{\prime }=0.338504{\left({0.5Re}_{\omega }\right)}^{0.5}\;\;\;\;\;\;\;\;\;\;\;\;\;\;{40\leq Re}_{R}\leq 100$$

where *Re_ω_* refers to vorticity Reynolds number, which can be calculated by Equation (19) below:

(19) $${Re}_{\omega }=\frac{\rho_{p}d_{R}^{2}\left|\nabla \times {\vec{u}}_{p}\right|}{\mu_{p}}$$

### 2.3. Geometry, Mesh and Boundary Conditions

**Figure 2:**
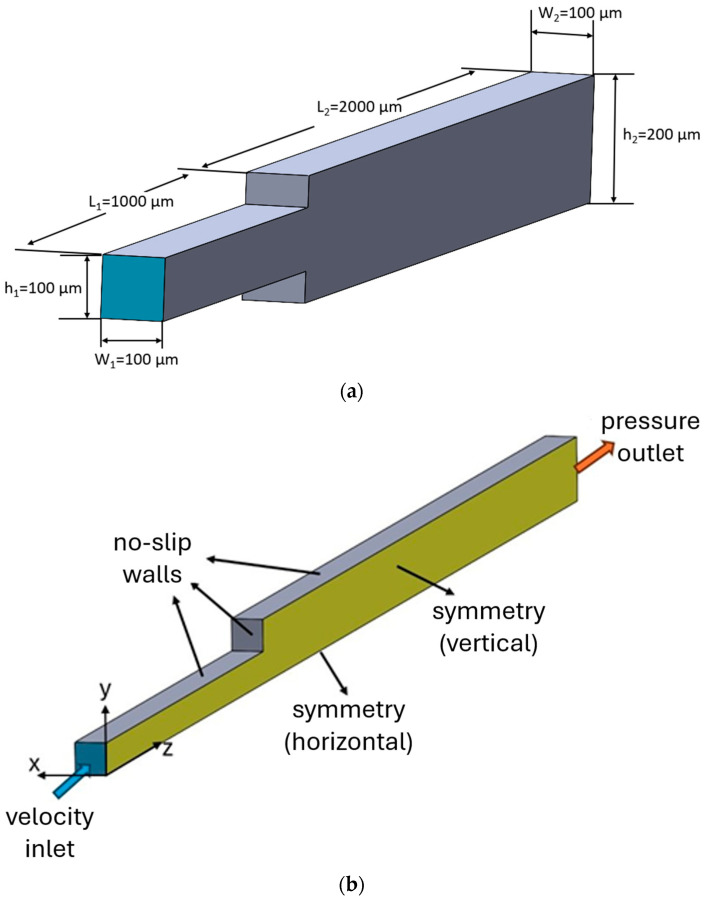
Sudden expansion microchannel: (**a**) full physical domain and dimensions, (**b**) the numerical model of the quarter-domain with the boundary conditions.

Figure 2a illustrates the physical geometry of the microchannel with a sudden expansion, which was previously studied by Zhao et al. [18] and Kim et al. [8]. The dimensions shown in Figure 2a correspond to the full physical domain. Accordingly, the microchannel consists of a narrow section with a length of *L*_1_ = 1000 µm and the wider section extending to *L*_2_ = 2000 µm. The cross-section of the narrow part measures *h*_1_ = 100 µm and *W*_1_ = 100 µm, while the wider section has the same width as that of the narrow section (*W*_1_ *= W*_2_) with a height of *h*_2_ = 200 µm. The flow enters the microchannel through the narrow section and exits through the wider section.

Figure 2b shows the numerical model employed in this study, where only one quarter of the physical domain is considered to reduce the computational cost by exploiting the geometric and flow symmetry about the vertical and horizontal mid-planes. Accordingly, symmetry boundary conditions are applied on the corresponding boundaries of the domain, which enforce the normal component of velocity and the normal gradients of all flow variables to be zero. A velocity inlet boundary condition is applied at the channel inlet, where uniform velocity and hematocrit profiles with fixed values are prescribed. Static pressure is set to zero-gauge pressure at the outlet by application of a pressure outlet boundary condition. No-slip boundary conditions are imposed on the microchannel walls. Table 2 presents a summary of the boundary conditions employed in the simulations.

A mesh independence test was performed for the representative case of *α_R_* = 0.2 and *d_R_* = 8 µm to ensure that the numerical results are independent of grid resolution. Three mesh configurations, i.e., coarse, medium and fine, were considered, consisting of approximately 0.8 × 10^6^, 1.6 × 10^6^, and 3.2 × 10^6^ hexahedral elements, respectively. Pressure drop values and RBC distribution profiles were compared among these meshes. The pressure drop deviation between coarse and medium meshes was found to be 0.65%, which further decreased to 0.02% for the fine mesh, as summarized in Table 3.

**Figure 3:**
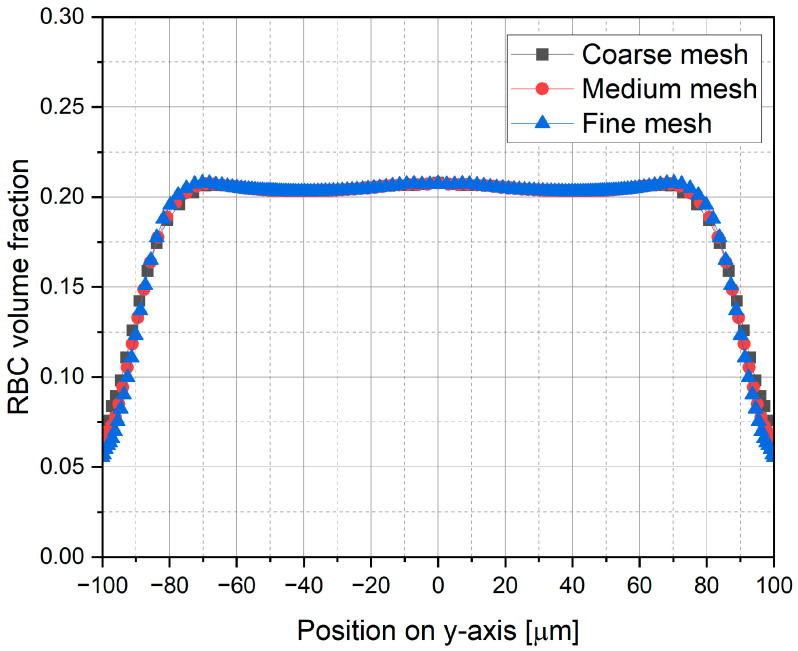
Comparison of mesh configurations in terms of RBC distribution along *y*-axis at *x* = 0 and *z* = 2000 μm (*α_R_* = 0.2 and *d_R_* = 8 µm).

**Figure 4:**
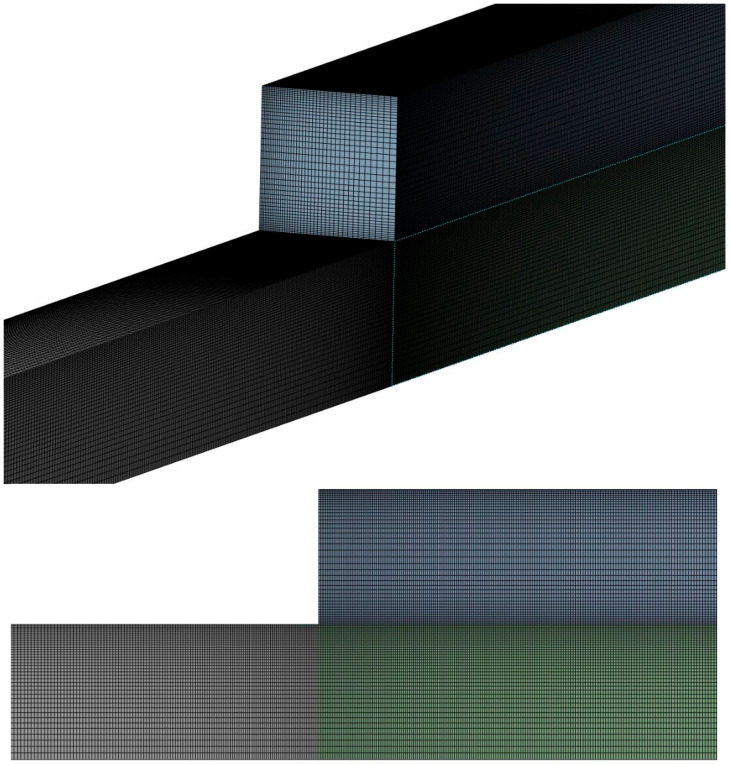
The grid used in numerical calculations.

In addition to pressure drop, RBC distribution profiles were used as a second criterion for mesh independence. Figure 3 presents the RBC volume fraction profiles along the *y*-axis at *x* = 0 and *z* = 2000 μm for all mesh configurations. The mean deviations in RBC volume fraction are 1.03% for the medium mesh and 0.76% for the fine mesh relative to the reference solution, as reported in Table 3. These results indicate that all mesh configurations capture the general trends of RBC migration and CFL formation, while improved quantitative agreement is achieved with the fine mesh refinement. Based on this analysis, the fine mesh configuration was selected for all subsequent simulations, which is illustrated in Figure 4.

### 2.4. Fluid Properties and Calculation Parameters

Table 4 summarizes microchannel dimensions, fluid properties and simulation conditions considered in this study, while calculation parameters are presented in Table 5. Accordingly, the effects of hematocrit at the channel inlet, RBC diameter, and blood flow rate on RBC distribution, CFL thickness, and *ΔP* in a microchannel with sudden expansion are investigated. The Reynolds number, calculated using the hydraulic diameter of the narrow channel section, inlet velocity, and plasma properties, remains within the laminar flow regime. Hydrodynamic entrance length (*L_e_*) is calculated using Equation (20) proposed by Ahmad and Hassan [36], which is valid for Reynolds numbers less than 500 and square microchannels with heights of 100, 200 and 500 μm. Accordingly, hydrodynamic entrance length is calculated as 172.67 μm, which corresponds to less than 20% of the length of the narrow part of the microchannel. Therefore, hydrodynamic entrance effects are considered negligible, and the flow is assumed to be fully developed.

(20) $$L_{e}=D_{h}\left(\frac{0.63}{0.035Re+1}+0.0752Re\right)$$

In the present study, deviations from the physiological RBC diameter of 8 μm are used to represent pathological alterations observed in various conditions, rather than explicitly modeling a specific disease with all its associated mechanisms. Diseases such as microcytic hypochromic anemia and hereditary spherocytosis are characterized by smaller RBCs, while, in general, enlarged RBCs are observed in cases of megaloblastic anemia, alcoholic liver disease and asthmatic bronchitis [26,27]. Accordingly, considering an RBC diameter of 8 μm as the reference corresponding to the healthy condition, RBC diameters of 4 and 11 μm are considered in simulations to investigate the effects of RBC size deviation from the representative healthy case on microscale blood flow behavior.

### 2.5. Calculation of the Cell-Free Layer

**Figure 5:**
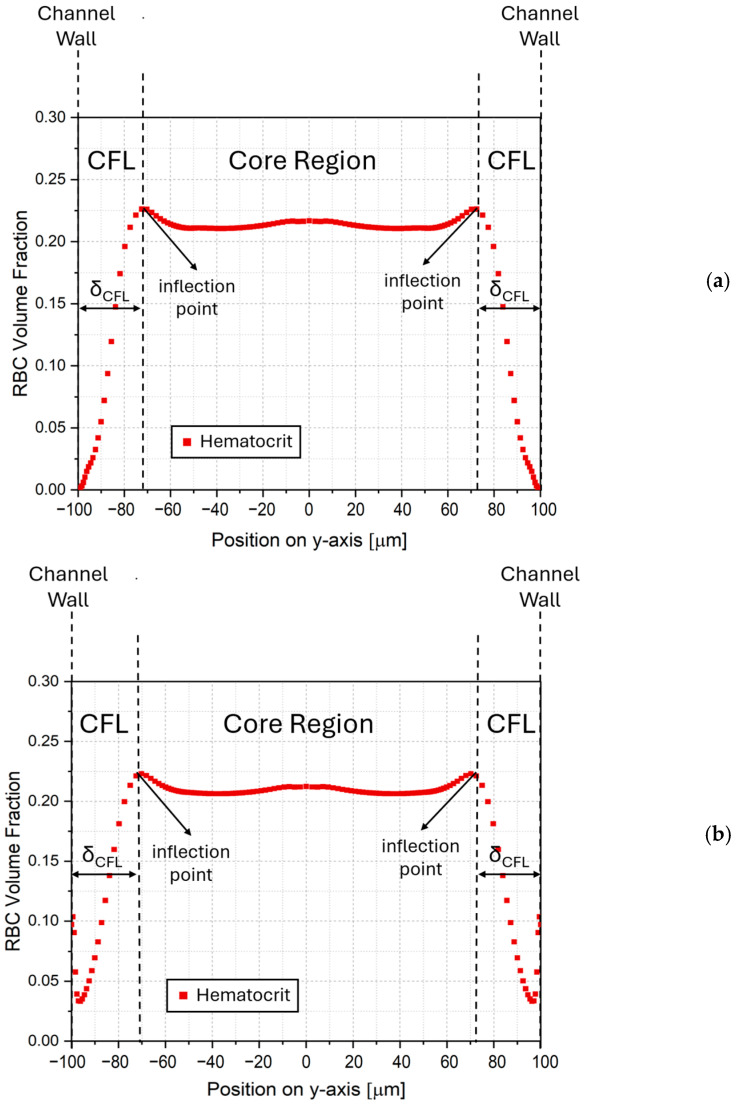
Sample hematocrit profiles and determination of the CFL thickness with (**a**) monotonic, and (**b**) non-monotonic profiles within the CFL.

Typical hematocrit profiles with monotonic and non-monotonic variation in the near-wall region, obtained from the numerical calculations are presented in Figure 5. Accordingly, two distinct regions are identified: a core region around the channel center where hematocrit has a nearly horizontal profile and a region near the wall with a sharp decrease in hematocrit, i.e., the CFL. Here, non-monotonic variation adjacent to the wall is considered a phenomenon in the CFL, rather than a separate region. Therefore, hematocrit data for |*y/h*| > 0.48 are excluded to avoid non-monotonic regions affecting the estimation, where |*y/h*| = 0 and |*y/h*| = 0.5 correspond to the channel centerline and the wall, respectively. In this study, the upper edge of the CFL is mathematically defined as the first inflection point moving away from the channel wall, within the region |*y/h*| < 0.48. Accordingly, the dimensionless CFL thickness ($\delta_{CFL}^{*}$) at a given cross-section is calculated by Equation (21), defined as the distance between the wall location ($y_{wall}$) and the first inflection point ($y_{inflection}$), normalized by the local channel height, *h*.

(21) $$\delta_{CFL}^{*}=\frac{\delta_{CFL}}{h}=\frac{y_{wall}-y_{inflection}}{h}$$

Through this approach, the formation of the CFL and the presence of RBC diminishing zones are systematically distinguished. Streamwise variation in CFL thickness values calculated using this approach is then presented in Section 3.4.

## 3. Results and Discussion

### 3.1. Validation of the Numerical Model

**Figure 6:**
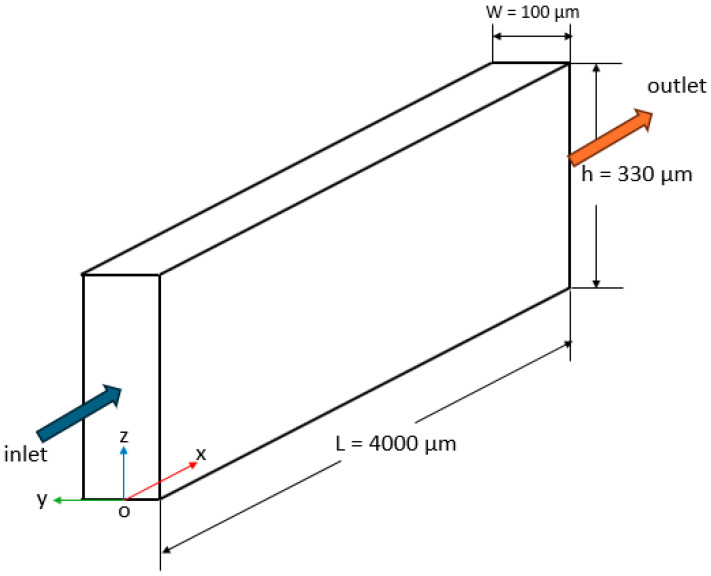
Rectangular microchannel used for the validation study.

**Figure 7:**
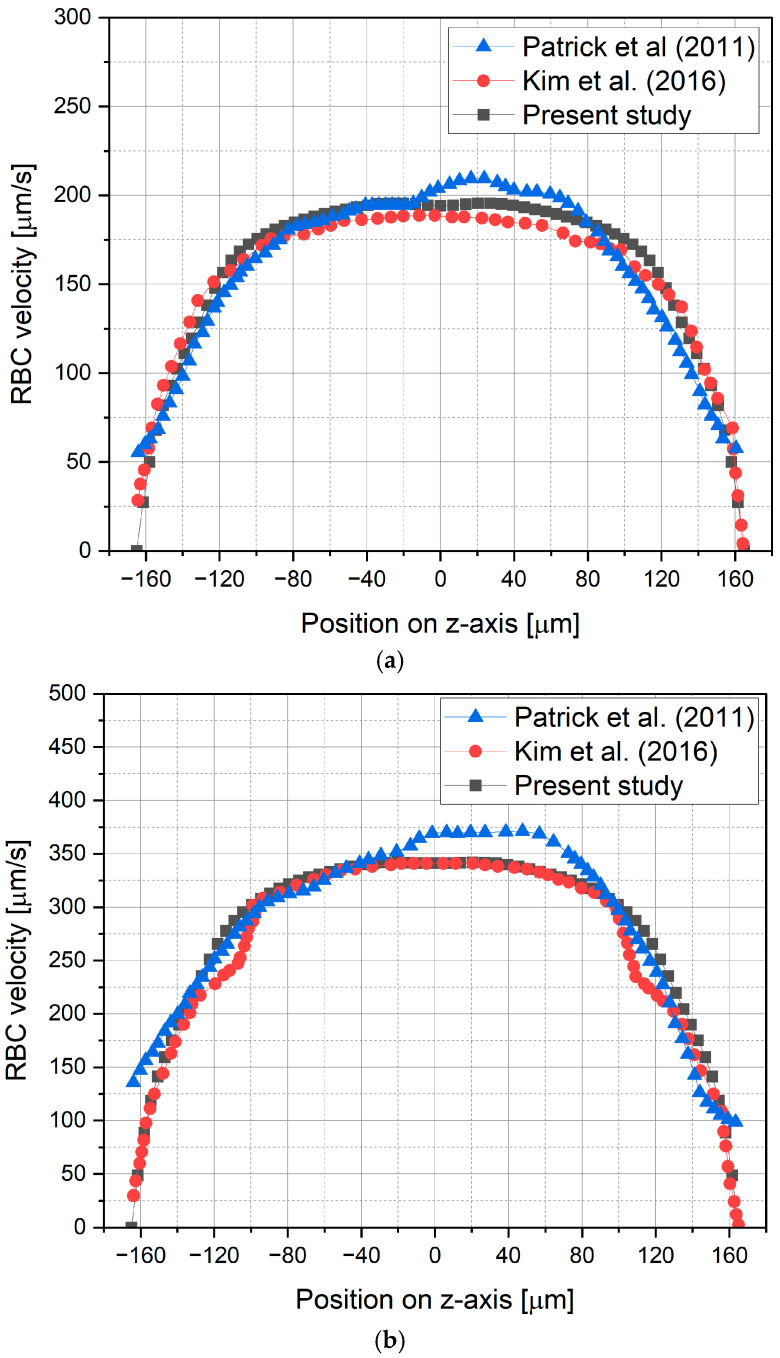
RBC velocity profiles along the spanwise locations of (**a**) 8 μm, and (**b**) 16 μm away from the wall at a cross-section of x = 2500 μm, compared with Patrick et al. [17] and Kim et al. [8].

**Figure 8:**
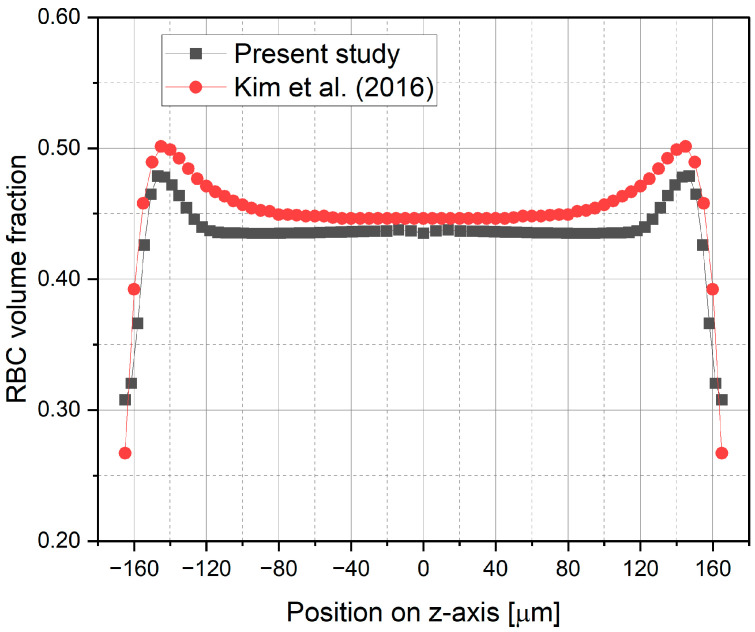
RBC volume fraction profiles plotted at the streamwise location of *x* = 2500 μm on the symmetry plane, in comparison with the results of Kim et al. [8].

To validate the numerical model used in this study, a rectangular microchannel (Figure 6) previously used in the experimental work of Patrick et al. [17] and the numerical study of Kim et al. [8] is considered. The validation is performed by comparing the obtained RBC velocity profiles and their distribution within the microchannel. Figure 7 illustrates a comparison of vertical RBC velocity profiles plotted at spanwise locations of 8 μm and 16 μm away from the wall, at a cross-section of *x* = 2500 μm. It is shown that the RBC velocity profiles obtained in this study are in good agreement with previous experimental and numerical studies conducted by Patrick et al. [17] and Kim et al. [8], respectively. In addition, the RBC volume fraction distributions evaluated at the streamwise location of *x* = 2500 μm on the symmetry plane are compared with those of the previous numerical study performed by Kim et al. [8], as shown in Figure 8. Accordingly, the region of diminishing RBC concentration near the wall and the migration of RBCs toward the core region of the microchannel are clearly captured by the present numerical calculations, as well as by Kim et al. [8], while the RBC concentration level at the core region and the location of its maximum show a good agreement.

### 3.2. Effect of Blood Flow Rate on RBC Distribution

**Figure 9:**
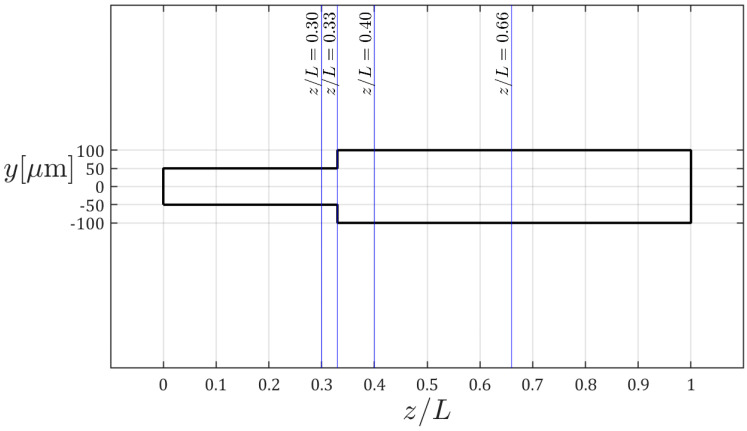
Cross-sectional locations at which RBC volume fraction data are plotted.

**Figure 10:**
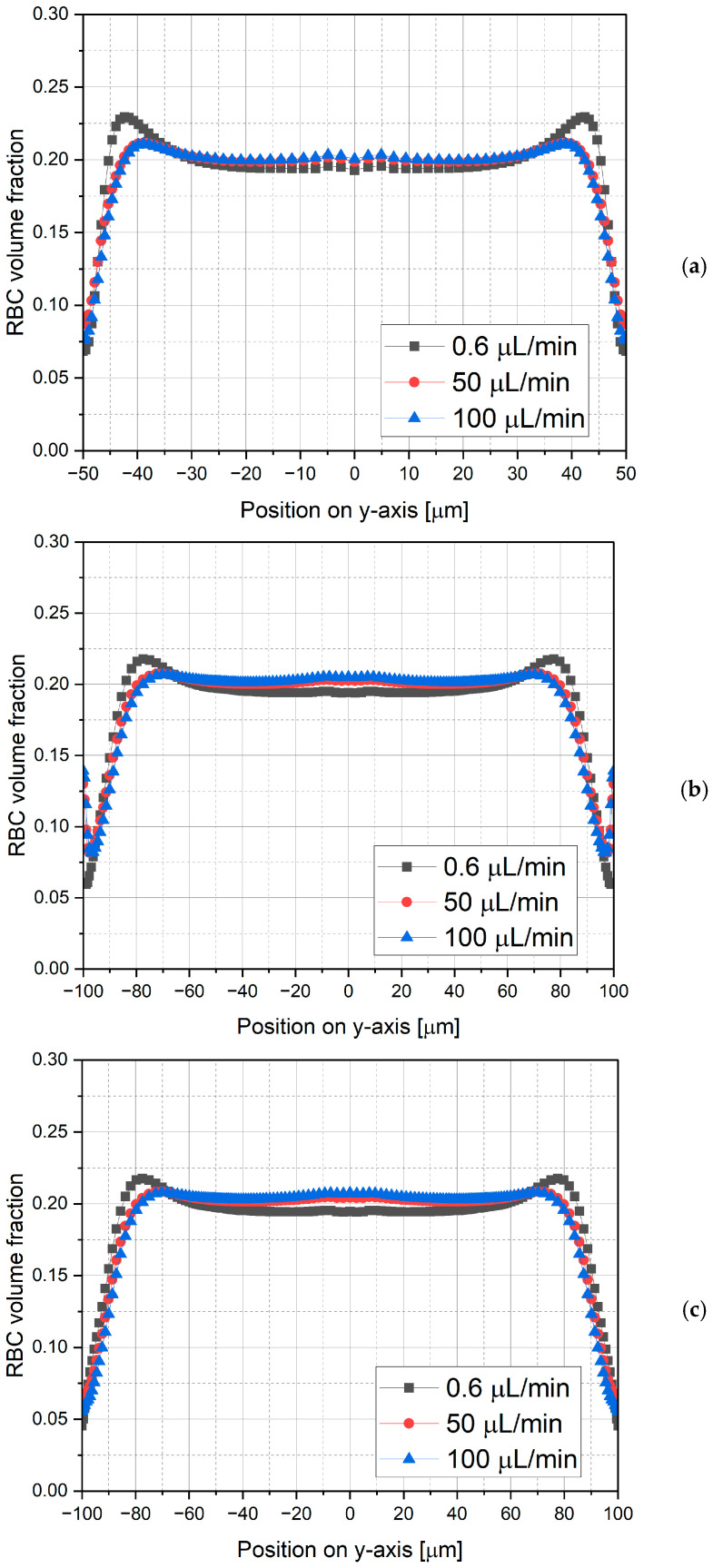
Cross-sectional profiles of RBC volume fraction for (**a**) *z/L* = 0.30, (**b**) *z/L* = 0.40 and (**c**) *z/L* = 0.66 (*α_R_* = 0.2 and *d_R_* = 8 μm).

The cross-sectional locations at which RBC volume fraction profiles are extracted are illustrated in Figure 9. The microchannel features a sudden expansion at *z*/*L* = 0.33, and the profiles are evaluated immediately upstream (*z*/*L* = 0.30) and downstream of the expansion (*z*/*L* = 0.40), as well as further downstream at *z*/*L* = 0.66. The corresponding RBC volume fraction distributions for various volumetric flow rates (0.6 μL/min, 50 μL/min, and 100 μL/min) at a constant inlet hematocrit of *α_R_* = 0.2 and RBC diameter of *d_R_* = 8 μm are presented in Figure 10. In general, hematocrit profiles follow the same trend for all volumetric flow rates considered. However, higher peak values are evident in the RBC volume fraction distributions at the lowest volumetric flow rate of 0.6 μL/min, which can be attributed to relatively lower shear rates and the resulting weaker shear-induced cell migration. At higher flow rates (50 and 100 μL/min), the RBC volume fraction profiles exhibit no significant variation throughout the channel. These results indicate that flow rate has a limited effect on RBC distribution once a sufficient shear rate is established. Accordingly, a volumetric flow rate of 100 μL/min is adopted for the remaining analyses, as it provides representative RBC distributions.

### 3.3. Effect of RBC Diameter and Hematocrit on RBC Distribution and CFL Thickness

**Figure 11:**
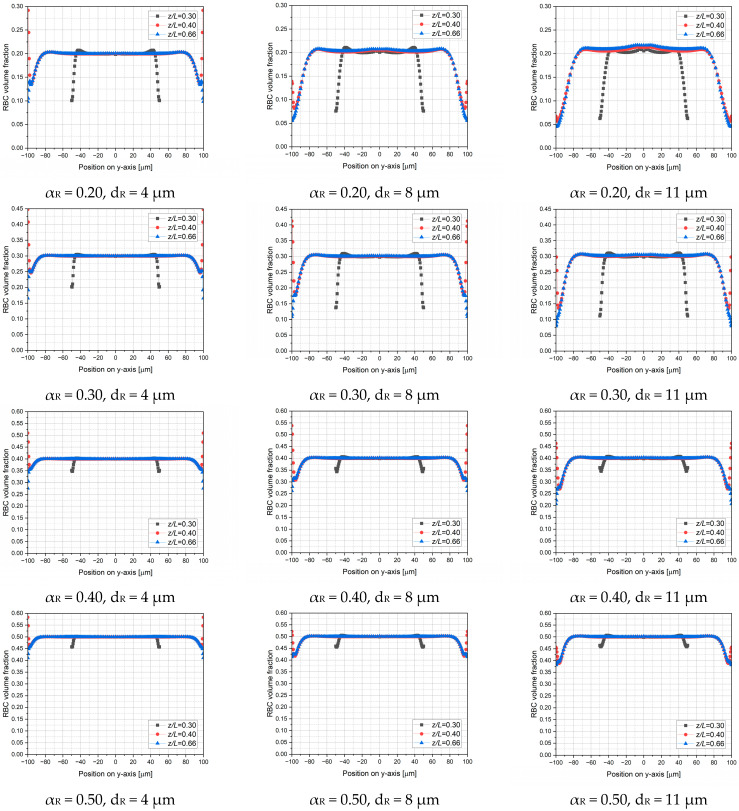
RBC distribution in the microchannel for diameters of 4 μm (microcytic anemia, spherocytosis), 8 μm (healthy), and 11 μm (megaloblastic anemia, alcoholic liver disease, asthmatic bronchitis).

**Figure 12:**
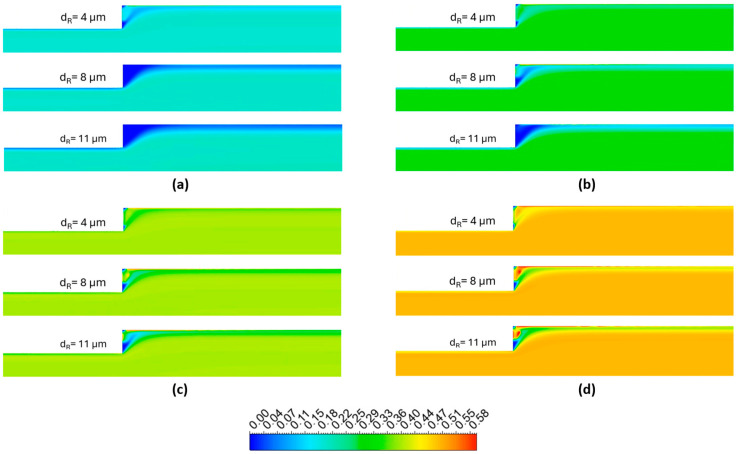
RBC distribution contours on the symmetry plane for hematocrit values of (**a**) α_R_ = 0.20, (**b**) α_R_ = 0.30, (**c**) α_R_ = 0.40 and (**d**) α_R_ = 0.50 for various RBC diameters corresponding to different pathological conditions.

Figure 11 and Figure 12 present a general picture of RBC distribution in the microchannel. Hematocrits of *α_R_* = 0.2, 0.3, 0.4 and 0.5 at the channel inlet and RBC diameters of *d_R_* = 4, 8 and 11 μm are considered, where *d_R_* = 8 μm represents the healthy reference, while deviations simulate pathological conditions, such as microcytic hypochromic anemia and hereditary spherocytosis for smaller RBCs, and megaloblastic anemia, alcoholic liver disease, and asthmatic bronchitis for enlarged RBCs.

Figure 11 illustrates the hematocrit profiles plotted at different streamwise locations (*z*/*L* = 0.30, 0.40 and 0.66) on the symmetry plane, while Figure 12 depicts contour plots of RBC distribution on the same symmetry plane. In Figure 12, the color scale represents the RBC volume fraction, where lower values correspond to the CFL, while higher values indicate regions of increased RBC concentration. Accordingly, RBC distribution strongly depends on both hematocrit and RBC size, which exhibit inverse effects owing to two distinct mechanisms. Increasing RBC size enhances shear-induced migration and lift effects, forcing RBCs to move towards the core region, which is characterized by lower shear rates. This migration causes the cell-depleted near-wall region to be more pronounced and consequently improves CFL formation, consistent with the Fahraeus–Lindqvist effect. However, an increase in hematocrit causes enhanced crowding of RBCs, yielding a densely populated core region that weakens cell migration. Therefore, at higher hematocrit levels, RBCs tend to penetrate the near-wall region, which is evident by elevated RBC concentrations close to the wall.

As shown in Figure 11, a well-defined CFL is observed upstream of the sudden expansion (*z*/*L* = 0.30) particularly at lower hematocrit values. In contrast, immediately downstream of the expansion (*z*/*L* = 0.40) the hematocrit profiles exhibit a non-monotonic behavior within the CFL region, indicating enhanced local mixing that increases the RBC volume fraction near the wall. Further downstream (*z*/*L* = 0.66), the effect of the recirculating flow gradually diminishes, and hematocrit profiles revert to monotonic distribution.

Figure 12 illustrates the distribution of RBC volume fraction on the symmetry plane for different hematocrit levels and RBC diameters. Accordingly, increase in the CFL thickness with RBC diameter is clearly visible. A distinct cell-depleted zone characterized by a locally reduced hematocrit develops immediately downstream of the expansion. This cell-depleted zone is distinguishable particularly at *α_R_* = 0.2 with normal and enlarged RBCs, and *α_R_* = 0.3 with enlarged RBCs only. However, a non-uniform cell-depleted zone is observed for the remaining cases. Consequently, although an increase in RBC size promotes the formation of a well-defined cell-depleted zone, higher hematocrit levels act in the opposite direction, in accordance with the mechanisms governing RBC migration.

**Figure 13:**
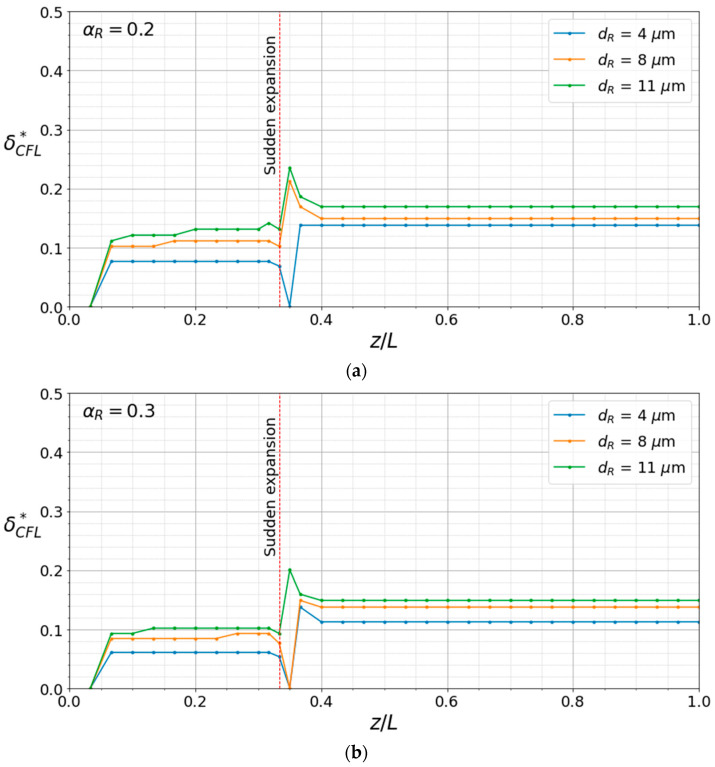

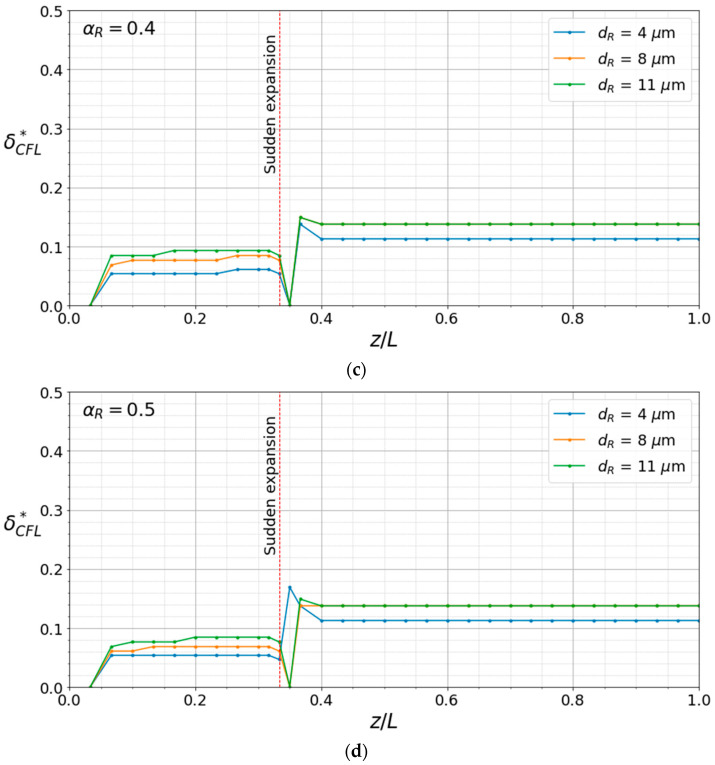
Streamwise variation in the dimensionless CFL thickness ($\delta_{CFL}^{*}$) within the microchannel for hematocrit of (**a**) α_R_ = 0.20, (**b**) α_R_ = 0.30, (**c**) α_R_ = 0.40, and (**d**) α_R_ = 0.50 for different RBC diameters.

Figure 13 illustrates the streamwise variation of normalized CFL thickness ($\delta_{CFL}^{*}$), which provides further evidence of the effect of cell size on RBC migration, justifying the fact that an increase in RBC size leads to a thicker CFL, as also shown in Figure 12. The opposite effect of hematocrit and RBC size on $\delta_{CFL}^{*}$ is also clearly observed in Figure 13. The sudden expansion at *z*/*L* = 0.33 leads $\delta_{CFL}^{*}$ to change dramatically immediately downstream of the expansion due to flow separation, resulting in local minima and maxima in that region, beyond which $\delta_{CFL}^{*}$ exhibits a nearly flat profile. Accordingly, thicker $\delta_{CFL}^{*}$ values are observed in the wider channel than in the narrow channel, which can be attributed to the presence of the sudden expansion.

These findings related to RBC distribution and CFL thickness variation suggest the dual influence of RBC size and hematocrit on the development of CFL in microscale hemodynamics, with potential implications for microscale transport phenomena, including shear-dependent diffusivity in microvascular environments.

### 3.4. Effect of RBC Diameter and Hematocrit on Pressure Drop

**Figure 14:**
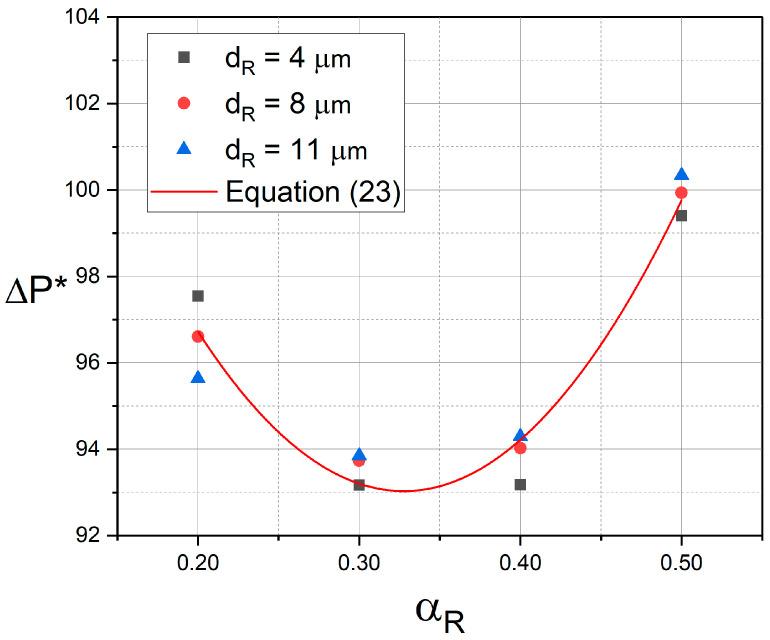
Variation in dimensionless pressure drop (∆P*) with hematocrit for *d_R_* = 4, 8 and 11 μm.

Figure 14 illustrates the variation in dimensionless pressure drop (∆P*) with hematocrit for different RBC sizes (*d_R_* = 4, 8 and 11 μm). The pressure drop is normalized by the plasma density (*ρ_p_*) and the inlet velocity (*u_i_*) (Equation (22)). The results indicate that the pressure drop is primarily governed by the hematocrit, in contrast to the apparent influence of RBC size on RBC distribution as explained in Section 3.3. While variation in RBC size has a significant impact on RBC distribution and CFL formation (Figure 11, Figure 12 and Figure 13), its effect on pressure drop remains negligible. Accordingly, ∆P* exhibits a maximum deviation of approximately 1% with varying *d_R_* for a constant *α_R_*, taking the healthy case (*d_R_* = 8 μm) as the reference. The minimum ∆P* is observed at *α_R_* = 0.3, indicating an optimal hematocrit value in terms of flow resistance. This hematocrit level coincides with the value reported by Piety et al. [37] to maximize oxygen-carrying capacity, suggesting that the present results regarding pressure drop are consistent with previously reported optimal hematocrit levels in terms of oxygen transport. A non-linear curve fitting of ∆P* as a function of *α_R_* yields the quadratic polynomial given by Equation (23), with a maximum relative error of 1.13% at *α_R_* = 0.2 with *d_R_* = 11 μm.

(22) $$∆P^{*}=\frac{∆P}{\frac{1}{2}\rho_{p}u_{i}^{2}}$$

(23) $$∆P^{*}=226.72\alpha_{R}^{2}-148.57\alpha_{R}+117.37$$

### 3.5. Limitations and Future Perspective

One of the primary simplifications in the current study is modeling RBCs as rigid spherical particles, neglecting their characteristic biconcave shape and deformation behavior. Although this assumption significantly reduces the computational cost, it should be noted that quantitative results related to RBC migration toward the channel center and CFL formation can be affected by deformability, which is known to have an effect on microflows [38]. This limitation may be particularly relevant downstream of the sudden expansion, where rapidly varying extensional and shear flow fields are present.

Additionally, pathological conditions are represented solely by deviations in RBC size, without a complete disease-specific modeling of RBC biomechanics. The primary objective of this study is to isolate the effects of RBC size variations corresponding to certain pathological conditions on cell distribution and CFL thickness along the microchannel. However, restricting the representation of pathological conditions to RBC size variations alone may limit the model’s ability to capture other disease-related mechanisms influencing CFL formation and pressure drop, such as altered deformability and shape transitions. Nevertheless, this simplification enables a systematic investigation of size-dependent effects without introducing additional modeling complexity.

Furthermore, the simulations are conducted under steady and laminar flow conditions to evaluate the mean characteristics of microscale blood flow. Although cell-induced temporal fluctuations may occur at high hematocrit levels and in the expansion region, recent experimental studies demonstrated that steady-flow simulations accurately capture the mean characteristics of the CFL. Unsteady flow conditions mainly influence temporal fluctuations without significantly altering the mean RBC distribution, CFL formation, or pressure drop [22].

Despite these limitations, the qualitative trends and the specific impact of size variations in RBCs reported in this study are expected to remain valid. Future work incorporating deformable RBC models, dynamic shape transitions, and unsteady flow conditions is expected to provide a more comprehensive understanding of the combined effects of size, shape and deformability on microscale hemodynamics. In addition, experimental studies in microchannels with geometric disturbances can be employed to obtain RBC distribution, CFL thickness and pressure drop under pathological conditions, providing further validation and refinement of the present numerical framework.

## 4. Conclusions

This study numerically investigates how pathological changes in RBC size and hematocrit alter microscale hemodynamics, focusing on pressure drop, RBC distribution and CFL thickness. The main conclusions can be summarized as follows:

- RBC size and hematocrit strongly govern RBC spatial distribution and CFL development.
- Larger RBCs exhibit enhanced migration toward the channel core, producing a thicker CFL.
- Smaller RBCs tend to remain closer to vessel walls, resulting in a thinner CFL.
- Increasing hematocrit reduces CFL thickness for all RBC sizes.
- Larger RBCs maintain a smoother cell-depleted layer immediately downstream of the sudden expansion at low to moderate hematocrit levels.
- Blood flow rate has a negligible effect on both RBC distribution.
- While RBC size significantly influences local cell distribution and CFL formation, its impact on the overall pressure drop remains limited.
- A non-linear relationship is evident between hematocrit (*α_R_*) and dimensionless pressure drop, ΔP*, where a parabolic trend is observed with a minimum ΔP* at *α_R_* = 0.3.
- The *α_R_* − ΔP* relationship is well represented by a second-order polynomial correlation, with a maximum relative error of 1.13% over the range 0.2 ≤ *α_R_* ≤ 0.5.
- The optimal hematocrit level of *α_R_* = 0.3 calculated by the present numerical calculations is compatible with the hematocrit corresponding to optimum oxygen transport efficiency, as reported previously.

In summary, the present results suggest that microscale blood flow is primarily governed by RBC size and hematocrit. Dependence of the CFL thickness, RBC distribution and pressure drop on these parameters indicates that pathological deviations may influence hydrodynamics of flow through microchannels significantly. Thus, the present study may serve as a useful guide for the development and optimization of microfluidic devices operating under non-ideal or disease-related conditions.

## Figures and Tables

**Table 1:** Literature survey on the investigation of blood flow in microchannels.

No	Researcher	Year	Methods	Key Findings
1	Gidaspow and Huang [3]	2009	Kinetic theory of granular flow in 2d narrow tube	Effectively explains the Fahraeus–Lindqvist effect/accurately predicts RBC distribution in narrow channels.
2	Kim et al. [8]	2016	Theory of interacting continua (mixture theory) with non-Newtonian viscosity model	Accurately estimates RBC depletion, particularly in the corners of the sudden expansion.
3	Gracka et al. [9]	2022	Euler–Euler approach with non-Newtonian viscosity model and Euler–Lagrange approach with Discrete Phase Model (DPM)	Simulates CFL formation and RBC dynamics in microchannels
4	Soh et al. [10]	2017	Volume of Fluid (VOF) Model	Simulates complex microscale flow and mass transfer phenomena cost-efficiently.
5	Jafari et al. [11]	2009	Fluid Structure Interaction (FSI) and Volume of Fluid (VOF) model	Provides valuable insights into the dynamic characteristics of blood flow in microvessels
6	Afzal and Kim [12]	2014	non-Newtonian viscosity model in straight and serpentine microchannels	Better mixing efficiency can be obtained using serpentine microchannel.
7	Barbosa et al. [13]	2023	Euler–Lagrange approach with Discrete Phase Model (DPM)	Accurately predicts RBC dynamics in hyperbolic contractions
8	Yin et al. [14]	2013	immersed-boundary lattice Boltzmann	The hematocrit phase separation has been reproduced in the simulations
9	Yaginuma et al. [15]	2013	Experimental in hyperbolic contraction microchannel	Microfluidic systems with hyperbolic-shaped microchannels offer a promising in vitro method for assessing RBC deformability and separating plasma.
10	Pinho et al. [16]	2016	Experimental in microtube	Temperature affects RBC distribution in microchannels.
11	Patrick et al. [17]	2011	Experimental in rectangular microchannel	Intermittent two-phase flow in high-hematocrit blood reveals unsteady RBC dynamics and validates computational models.
12	Zhao et al. [18]	2008	Experimental in sudden expansion microchannel	RBC volume fraction influences formation of CFL in sudden expansion microflows, with higher RBC volume fraction enhancing particle concentration in flow separation regions.
13	Lee et al. [19]	2009	Experimental in hyperbolic contraction microchannel	The new microchannel device was found to be more efficient in inducing cell deformation compared to the shear flow.

**Table 2:** Boundary conditions.

Boundary Type	Applied Condition
Inlet	Fixed velocity and volume fraction
Outlet	Fixed static pressure (0 Pa)
Wall	No-slip wall
Symmetry	Zero normal velocity and zero normal gradients for all variables

**Table 3:** Comparison of mesh configurations in terms of pressure drop and RBC volume fraction (*α_R_* = 0.2 and *d_R_* = 8 µm).

Mesh Configuration	Number of Elements	Pressure Drop [Pa]	Variation in Pressure Drop [%]	Mean Deviation in RBC Volume Fraction [%]
Coarse	0.8 × 10^6^	1374.9	-	-
Medium	1.6 × 10^6^	1383.8	0.65	1.03
Fine	3.2 × 10^6^	1383.5	0.02	0.76

**Table 4:** Microchannel dimensions, fluid properties and simulation conditions.

Microchannel dimensions	
*h*_1_	100 μm
*W*_1_	100 μm
*L*_1_	1000 μm
*h*_2_	200 μm
*W*_2_	100 μm
*L*_2_	2000 μm
Fluid properties	
*ρ_p_*	1027 kg/m^3^
*ρ_R_*	1093 kg/m^3^
*μ_p_*	0.00096 kg/m s
*μ_R_*	Calculated by a Carreau–Yasuda-type viscosity model
*λ*	0.110 s
Simulation conditions	
Solution type	Steady-state
Flow regime	Laminar

**Table 5:** Calculation parameters.

Inlet Velocity [m/s]	Volumetric Flow Rate [μL/min]	Re	RBC Volume Fraction at the Inlet (*α_R_*)	RBC Diameter (*d_R_*) [μm]
0.0010 0.0835 0.1670	0.6 50 100	0.11 8.90 17.80	0.2 0.3 0.4 0.5	4 8 11

## Data Availability

The datasets used and analyzed during the current study are available from the corresponding author on reasonable request.

## Nomenclature

The following abbreviations are used in this manuscript:

Symbols	
*α_p_*	Plasma volume fraction
*α_R_*	RBC volume fraction (hematocrit)
β	Interphase momentum exchange coefficient (kg/m^3^ s)
$\dot{\gamma }$	Shear rate (s^−1^)
$\delta_{CFL}$	CFL thickness (μm)
$\delta_{CFL}^{*}$	Normalized CFL thickness
*η*	Dimensionless relative mixture viscosity
λ	Time constant (s)
μ	Dynamic viscosity (kg/m s)
ρ	Density (kg/m^3^)
τ	Stress (N/m^2^)
*C_D_*	Interphase drag coefficient
*C_l_*	Interphase lift coefficient
*D_h_*	Hydraulic diameter (μm)
*d_R_*	RBC diameter (μm)
${\vec{F}}_{drag}$	Interphase drag force (N/m^3^)
${\vec{F}}_{lift}$	Interphase lift force (N/m^3^)
*h*	Channel height (μm)
*L*	Channel length (μm)
*L_e_*	Hydrodynamic entrance length (μm)
*m, n*	Carreau–Yasuda-type viscosity model coefficients
*ΔP*	Pressure drop (Pa)
*ΔP**	Normalized pressure drop
*Re_ω_*	Vorticity Reynolds number
*Re_R_*	RBC Reynolds number
*u*	Velocity (m/s)
*W*	Channel width (μm)
Abbreviations	
CFL	Cell-free layer
RBC	Red blood cell
Subscripts	
*mix*	Mixture
*p*	Plasma
*R*	Red Blood Cell (RBC)
1, 2	Channel indices for upstream and downstream of the expansion
